# Predicting stroke-associated infection in acute ischemic stroke patients treated by thrombolysis

**DOI:** 10.3389/fncel.2026.1761927

**Published:** 2026-03-10

**Authors:** Xuanyue Yu, Zeyuan Wang, Dong Chen, Shuming Li, Haojie Gao, Wen Zhao, Zishan Ji, Ziqi Han, Ruikang Sun, Shuya Cai, Zhicheng Jiang, Shiwei Du, Dirk M. Hermann, Yi Liu

**Affiliations:** 1 Department of Neurology, Central Hospital of Dalian University of Technology, Dalian, China; 2 School of Control Science and Engineering, Dalian University of Technology, Dalian, China; 3 Department of Neurosurgery, Central Hospital of Dalian University of Technology, Dalian, China; 4 Institute of Cardio-Cerebrovascular Medicine, Dalian University of Technology, Dalian, China; 5 Dalian Medical University, Dalian, China; 6 Department of Neurosurgery, South China Hospital of Shenzhen University, Shenzhen, China; 7 Department of Neurology, University Hospital Essen, University of Duisburg-Essen, Essen, Germany

**Keywords:** prediction model, prevention, prognosis, risk factor, Rt-PA, stroke

## Abstract

**Background:**

Acute ischemic stroke (AIS) remains one of the major contributors to mortality and disability worldwide. Stroke-associated infection (SAI) is one of the most frequent complications following AIS and has a substantial impact on clinical outcomes, being closely linked to unfavorable prognosis. This study aimed to provide a comprehensive description of SAI, identify independent risk factors, and develop a predictive nomogram for its early identification.

**Methods:**

This study included 836 AIS patients of the Dalian Single-center Study on Intravenous Thrombolysis for Ischaemic Stroke (DATIS) cohort who received recombinant tissue-plasminogen activator-induced thrombolysis at Central Hospital of Dalian University of Technology between January 2018 and November 2021. Patients were divided into a training cohort (*n =* 586, 70%) and a validation cohort (*n =* 250, 30%). Composition and economic features of SAI was explored. Independent risk factors were identified using univariate, multivariate, and multimodal logistic regression analyses. A predictive nomogram was then developed based on these independent risk factors. Model performance was assessed with receiver operating characteristic curves, and calibration curves.

**Results:**

Among the 836 enrolled patients, 168 (20.1%) developed SAI. Composition of 168 patients with SAI were: 99 pulmonary infections (58.93%), 44 upper respiratory tract infections (26.19%), 15 urinary tract infection (8.93%), 2 gastrointestinal tract infections (1.19%), 1 periodontal infection (0.60%), 1 conjunctival infection (0.60%), and 1 erysipela (0.60%). In addition, 5 patients (2.98%) had multi-site infections (4 pulmonary plus urinary tract infection, 1 pulmonary plus gastrointestinal tract infection). Compared with non-infected patients, the SAI group experienced a significantly longer median hospitalization duration [9 days, IQR (7, 10) vs. 8 days, IQR (7, 9), *p <* 0.001] and incurred higher median inpatient medical costs [28114.04 RMB, IQR (23230.12, 33379.85) vs. 22292.84 RMB, IQR (19203.53, 25999.63), *p <* 0.001]. Five variables—higher modified Rankin Scale at admission, male sex, prolonged prothrombin time, elevated blood urea nitrogen and lower thyroid-stimulating hormone—were independent risk factors for SAI. The nomogram constructed based on above predictors achieved an area under the curve of 0.80 in the training cohort and 0.72 in the validation cohort. Calibration curves supported the model’s performance.

**Conclusion:**

This prospective cohort study comprehensively described composition and economic features, identified risk factors and developed predictive nomogram for SAI in AIS patients receiving intravenous rt-PA. Early identification of high-risk patients may facilitate targeted interventions, potentially reducing infection-related complications and improving clinical outcomes.

## 1 Introduction

Acute ischemic stroke (AIS) stands as one of the leading causes of disability and death worldwide. Intravenous recombinant tissue-type plasminogen activator (rt-PA) has been approved as the first-line therapy for AIS in the United States, Europe, and China. However, stroke-associated infection (SAI), a common complication of AIS, has been shown to significantly worsen patient prognosis (Rocco et al., 2013). Although several studies focussed on the immune responses predisposing to SAI - immunodepression was found to play a decisive role (Tuz et al., 2024; Roth et al., 2021), the clinical factors predisposing to SAI are still not well defined. Hospital-associated infections are associated with prolonged hospitalization, exacerbation of pre-existing medical conditions, and impaired functional recovery (Fluck et al., 2024), highlighting the critical need for early prediction and prevention of this complication.

The incidence of post-stroke infections has been reported to reach up to 30% (95% CI 24–36%), with pulmonary infections (10, 95% CI 9–10%) and urinary tract infections (UTI) being the most common types (Westendorp et al., 2011). Other types of infections, such as upper respiratory tract, gastrointestinal, periodontal, conjunctival, and skin (erysipelas) infections, have also been reported in AIS patients, although less frequently (Fluck et al., 2024; Grau et al., 2004). Predictive studies have also focused mostly on stroke-associated pneumonia (SAP) and UTI. Clinical score, e.g., Ischemic Stroke-Associated Pneumonia, and machine learning were developed specifically for SAP prediction (Suda et al., 2018a; Smith et al., 2015; Xie et al., 2025). Post-stroke dysphagia, which was considered main contributor to SAP, was confirmed to have risk factors of older age, higher NIHSS, and right-hemispheric stroke (Krekeler et al., 2024). Prediction for UTI has been focused on severe AIS and associated with Foley catheter retention (Jitpratoom and Boonyasiri, 2023). Furthermore, epidemiologic investigations such as that by Krekeler et al. (2024) have identified risk factors for post-stroke dysphagia (a known contributor to respiratory infection).

Currently, comprehensive studies elucidating composition and economic characteristics of SAI - including but not limited to SAP and UTI - and developing clinically practical prediction model are scarce. This prospective cohort aims to elucidate SAI with more clinical details, identify independent risk factors for SAI, and to develop a nomogram prediction model, which enables individualized risk assessment at the bedside. Consequently, it facilitates to support early identification of high-risk individuals, to optimize post-thrombolysis monitoring, and ultimately to improve patient outcomes by enabling timely interventions.

## 2 Materials and methods

### 2.1 Study design and participants

This study includes AIS patients who received intravenous thrombolysis with rt-PA and were admitted to the Department of Neurology of Central Hospital of Dalian University of Technology between January 2018 and November 2021. This study is part of the prospective Dalian Single-center Study on Intravenous Thrombolysis for Ischaemic Stroke (DATIS) cohort study that is continuously recruited at the Central Hospital. The Central Hospital of Dalian University of Technology is a major hospital in the city of Dalian, an 8 million inhabitant city in the North-East of China. It therefore has broad access to AIS patients. The study protocol has been registered at ChiCTR2400089803.

The inclusion criteria were as follows: Admission between January 2018 and November 2021; age ≥ 18 years; confirmed diagnosis of AIS by a neurologist in accordance with the Chinese Guidelines for the Diagnosis and Treatment of Acute Ischemic Stroke issued in 2023 (Liu et al., 2023); according to the indications for intravenous thrombolysis, the patients and his family signed the informed consent for thrombolysis and received intravenous thrombolysis with rt-PA.

The exclusion criteria were as follows: Presence of infection within 3 days before stroke onset; taking antibiotics, steroids, immunosuppressants and other drugs before admission; bridging therapy with intravenous rt-PA combined with subsequent endovascular therapy; comorbid tumors and immune system disorders; pregnant women, lactating and preparing for pregnancy; incomplete clinical data.

Clinical judgment of SAI: The disease was diagnosed as acute ischemic stroke by a clinical physician, and infections involving any organ system developed that occurred during hospitalization after disease onset. The diagnostic criteria for SAI were based on internationally accepted standards for defining healthcare-associated infections, specifically the CDC’s National Healthcare Safety Network criteria (Horan et al., 2008; Garner et al., 1988).

### 2.2 Data collection

A comprehensive panel of 42 clinical and biochemical variables was collected at admission, covering demographic characteristics, neurological symptoms and impairment, vascular risk factors, and routine laboratory indices (hematologic, coagulation, metabolic, renal, hepatic, and thyroid parameters). The complete list and descriptive statistics of these variables are presented in Table 1 (Baseline Characteristics).

**Table 1:** Baseline characteristics: training vs. validation cohorts.

Variables	Training cohort (*n =* 586) Median (P25, P75)/*N* (%)	Validation cohort (*n =* 250) Median (P25, P75)/*N* (%)	*p*-value
NIHSS at admittance	4 (2, 7)	4 (2, 7)	0.632
mRS at admittance	1 (1, 2)	1 (1, 2)	0.246
Number of days of hospitalization	8 (7, 9)	8 (7, 10)	0.521
Age, years	68 (60, 78)	69 (60, 78)	0.888
Sex, no (%)			
Man	372	166	0.420
Women	214	84	
Smoking (%)	40.1	44.4	0.248
Alcoholism (%)	27.3	27.6	0.930
Medical history (%)			
Atrial fibrillation	16.7	19.2	0.388
Arterial hypertension	77.9	77.2	0.802
Diabetes	30.3	24.0	0.350
Coronary heart disease	16.3	18.8	0.395
Dyslipidemia	38.7	37.2	0.675
Previous stroke	13.4	12.4	0.672
Systolic blood pressure at admittance (mmHg)	159 (144, 173)	161 (144, 174)	0.411
Diastolic blood pressure at admittance (mmHg)	86 (80, 94)	88 (80, 96)	0.094
Stroke subtype based on TOAST			0.523
Large-artery atherosclerosis	257	111	
Cardioembolism	135	45	
Small-vessel occlusion	82	38	
Stroke of other determined etiology	85	42	
Stroke of undetermined etiology	27	14	
Location of responsible vessel			0.739
Anterior circulation	408	174	
Posterior circulation	110	43	
Anterior and posterior circulation	68	33	
Biochemical variables			
High density lipoprotein (HDL)	1.05 (0.89, 1.22)	1.04 (0.87, 1.23)	0.544
Apolipoprotein A1 (Apo A1)	1.25 (1.12, 1.38)	1.25 (1.12, 1.44)	0.557
Apolipoprotein B (Apo B)	0.94 (0.79, 1.09)	0.95 (0.82, 1.10)	0.729
Lipoprotein (a) (Lp(a))	181.50 (81.00, 345.25)	163.50 (72.50, 325.25)	0.222
Homocysteine (HCY)	13.5 (11.0, 17.2)	13.6 (11.0, 17.6)	0.679
White blood count (WBC)	7.12 (5.95, 8.69)	7.29 (6.04, 8.89)	0.422
Neutrophils (NEUT)	4.33 (3.50, 5.78)	4.50 (3.45, 5.80)	0.789
Lymphocyte (LYM)	1.90 (1.36, 2.52)	1.91 (1.50, 2.55)	0.328
Neutrophil/Lymphocyte (NLR)	2.21 (1.60, 3.52)	2.23 (1.52, 2.39)	0.452
Prothrombin time (PT)	13.0 (12.5, 13.6)	13.0 (12.4, 13.6)	0.858
Activated partial thromboplastin time (APTT)	35.6 (32.4, 39.4)	35.8 (32.7, 39.5)	0.455
Fibrinogen	3.28 (2.86, 3.72)	3.17 (2.79, 3.68)	0.088
Glucose	6.77 (5.73, 8.92)	6.76 (5.60, 8.43)	0.369
Blood Urea Nitrogen (BUN)	6.27 (5.20, 7.52)	6.45 (2.26, 7.86)	0.121
Creatinine clearance (Ccr)	67.0 (56.0, 79.0)	69.0 (57.0, 81.3)	0.198
Glomerular filtration rate (GFR)	99.21 (82.80, 120.05)	99.19 (89.93, 117.68)	0.602
Alanine aminotransferase (ALT)	17.0 (12.0, 24.0)	16.0 (12.0, 26.0)	0.576
Aspartate aminotransferase (AST)	19.0 (15.0, 24.0)	19.0 (15.0, 24.0)	0.907
Gamma-glutamyl transpeptidase (GGT)	23.0 (16.0, 36.0)	24.0 (18.0, 38.0)	0.303
Free triiodothyronine (FT3)	4.42 (3.87, 4.89)	4.49 (3.83, 5.00)	0.543
Free thyroxine (FT4)	15.02 (13.60, 16.65)	15.44 (13.94, 16.86)	0.204
Thyroid stimulating hormone (TSH)	1.33 (0.76, 2.13)	1.34 (0.79, 2.49)	0.524
Post-stroke infection	19.6	21.2	0.603
BMI	25.06 (23.1, −27.34)	25.06 (23.15, 27.05)	0.599

This figure presents the baseline characteristics of patients in the training cohort (*n =* 586) and validation cohort (*n =* 250). Data include demographic variables such as age, sex, and medical history, as well as clinical variables including NIHSS, mRS, hospitalization duration, and biochemical variables and indices. The data are presented as medians (P25, P75) and percentages, with *p*-values for group comparisons. Significant differences between the two cohorts are highlighted. SAI, stroke-associated infection; NI, no infection.

### 2.3 Statistical analysis

Quantitative data following a normal distribution were compared using the *t*-test and expressed as mean ± standard deviation. Non-normally distributed data were compared using non-parametric rank-sum tests and expressed as medians and interquartile ranges. Categorical variables are expressed as counts and percentages and compared using the chi-square test. Univariate logistic regression was performed to screen potential risk factors for SAI, and variables with *p <* 0.05 were entered into a multivariate logistic regression model to identify independent predictors (reported as ORs with 95% CIs). To further assess robustness, a series of progressively adjusted multimodal logistic regression models were constructed. Model 1 included basic demographic and lifestyle factors: age, sex, smoking, alcohol use, hypertension, diabetes, and dyslipidemia. Model 2 added vascular comorbidities, including coronary heart disease and previous stroke, to the variables in Model 1. Model 3 further incorporated neurological severity and laboratory markers: National Institutes of Health Stroke Scale (NIHSS) at admission, mRS at admission, homocysteine (HCY), white blood cell count (WBC), neutrophil count (NEUT), neutrophil-to-lymphocyte ratio (NLR), PT, BUN, creatinine clearance (Ccr), aspartate aminotransferase (AST), free triiodothyronine (FT3), TSH. A nomogram was constructed from the final model to predict individual SAI risk, incorporating an optimal cut-off point (determined by maximizing the Youden index on the ROC curve) for risk stratification. Model performance was evaluated by discrimination AUC, and calibration (calibration plot and Hosmer-Lemeshow test). SAI was further categorized into pulmonary, urinary tract, multisite, and other infections for descriptive analysis. Analyses were conducted using SPSS 26.0 and R software, with two-sided *p <* 0.05 considered significant.

## 3 Results

### 3.1 Patient selection and cohort division

**Figure 1:**
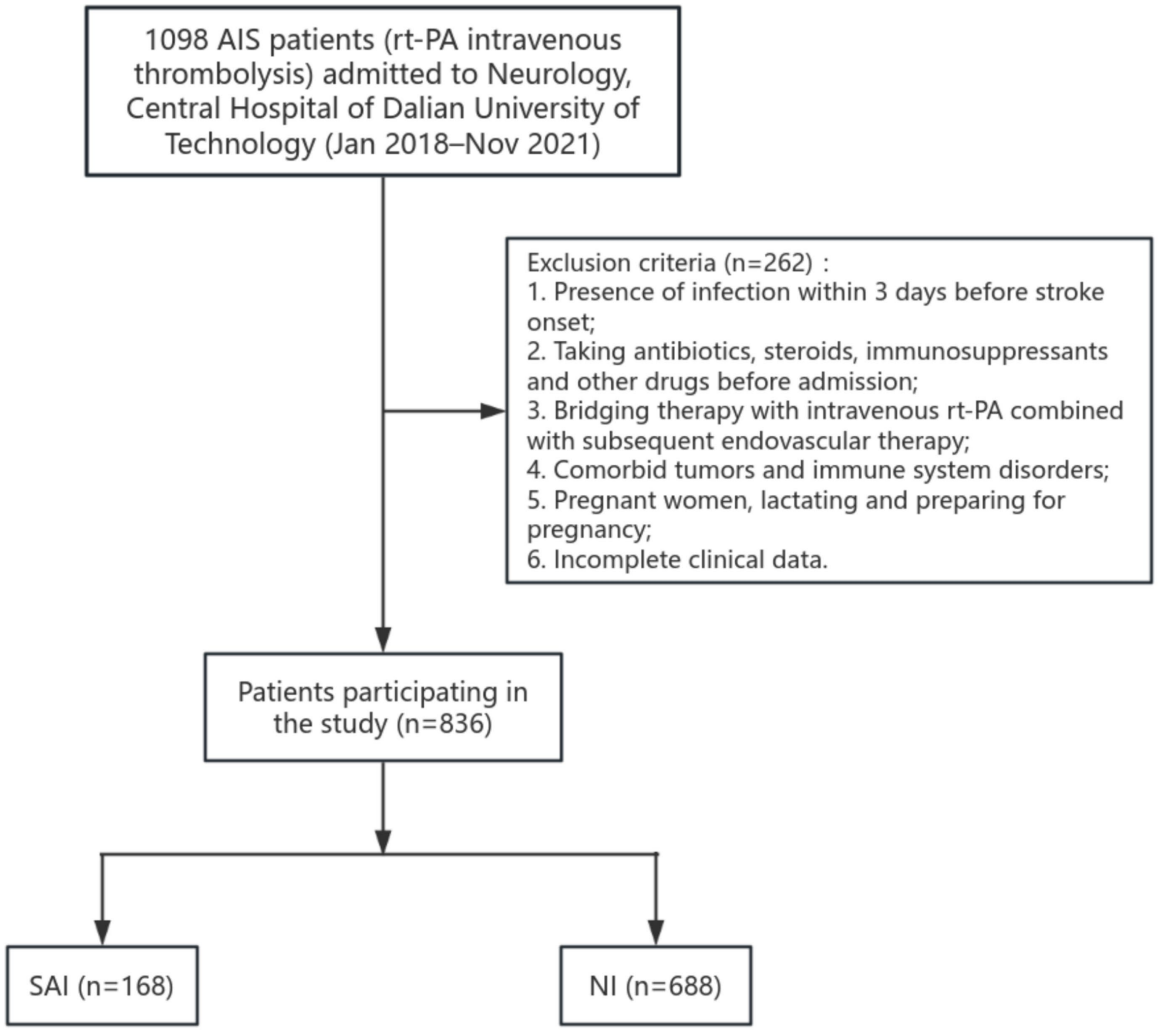
Flow diagram of patient enrollment for SAI in rt-PA-treated AIS patients. SAI, Stroke-associated infection; NI, No infection; rt-PA, Recombinant tissue-type plasminogen activator.

A patient categorization flowchart was created, depicting the enrollment of 836 patients receiving rt-PA-induced thrombolysis in the study (Figure 1). These patients were divided into a training cohort (*n =* 586, 70%) and a validation cohort (*n =* 250, 30%) at a ratio of 7:3. The training cohort was utilized for developing the prediction model, while the validation cohort was employed for model validation.

### 3.2 Clinical and economic features of SAI

#### 3.2.1 Composition and distribution of SAI

**Figure 2:**
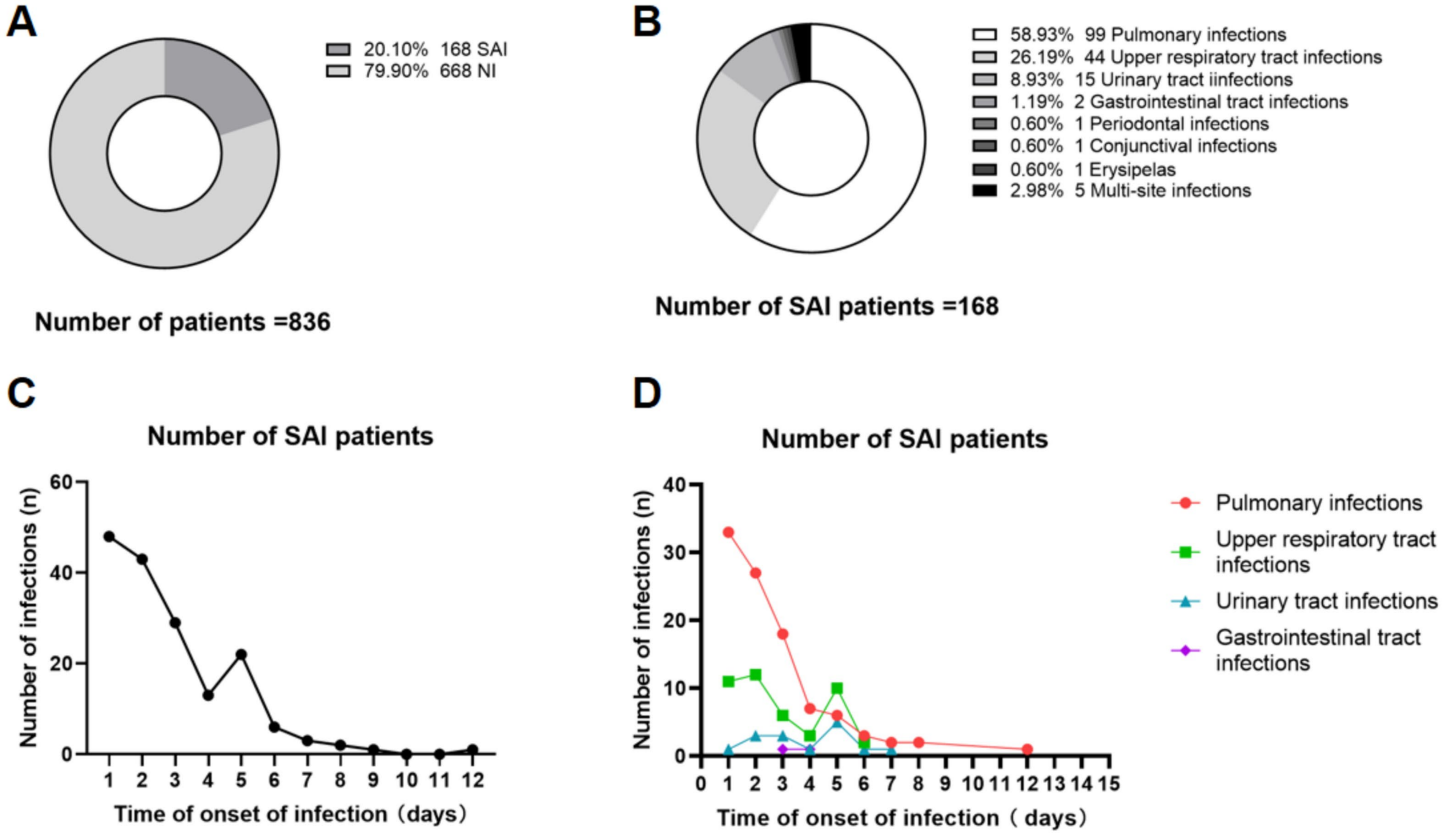
Composition and temporal dynamics of SAI in rt-PA-treated AIS patients. This figure presents the composition and temporal dynamics of SAI in rt-PA-treated AIS patients. **(A)** Shows the proportion of SAI and NI patients in the overall cohort of 836 patients. **(B)** Illustrates the distribution of infections types among 168 SAI patients, and multi-site infections (5, 2.98%). The 5 patients with multi-site infections included 4 cases of pulmonary infections combined with urinary tract infections, and 1 case of pulmonary infections combined with gastrointestinal tract infections. **(C)** Depicts the daily number of new SAI cases from admission (Day 1, defined as hospital admission) throughout the hospitalization period. **(D)** Illustrates the temporal dynamics of specific infection types (pulmonary, upper respiratory tract, urinary tract, and gastrointestinal tract), plotting the daily count of new cases against time post-admission (Day). SAI, Stroke-associated infection; NI, no infection; rt-PA, recombinant tissue-type plasminogen activator.

In this study, a total of 836 patients were enrolled, with 668 patients (79.1%) presenting no infection (NI) and 168 patients (20.10%) with confirmed SAI during hospitalization, as illustrated in Figure 2A. Composition of 168 patients with SAI were: 99 patients with pulmonary infection (58.93%), 44 patients with upper respiratory tract infection (26.19%), 15 patients with UTI (8.93%), 2 patients with gastrointestinal tract infection (1.19%), 1 patient with periodontal infection (0.60%), 1 patient with conjunctival infection (0.60%), 1 patient with erysipela (0.60%) and 5 patients with multi-site infection (2.98%), including 4 patients with pulmonary infection plus UTI and 1 patient with pulmonary infection plus gastrointestinal tract infection (Figure 2B).

As shown in Figure 2C, among 168 SAI patients, the total number of infections presented a distinct temporal pattern. The number of infections was the highest on day 1 (0–24 h after admission), with 48 cases, then decreased steadily to 13 cases on day 4, followed by a slight rebound to 22 cases on day 5. After day 5, the number of infections continued to decline, maintaining a low level (<5 cases) from day 8 onwards. The dynamic changes in the number of SAI patients with different infection types over the disease course are shown in Figure 2D. Pulmonary infection was the most common type in the early stage (day 1), with 33 cases, followed by a gradual decrease. Upper respiratory tract infection showed a fluctuating trend, peaking at day 1 (11 cases) and day 5 (10 cases). Urinary tract infection and gastrointestinal tract infection occurred less frequently, with the highest number of cases being 5 and 1, respectively, and remained at low levels throughout the observation period.

#### 3.2.2 Length of hospital stay and economic assessment

For length of hospital stay and economic outcomes, the SAI group had both a significantly longer median hospitalization duration [9 days, IQR (7, 10)] and higher median inpatient costs [28114.04 RMB, IQR (23230.12, 33379.85)] compared with the NI group [8 days, IQR (7, 9); 22292.84 RMB, IQR (19203.53, 25999.63)], with *p <* 0.001 for both comparisons (Table 2).

**Table 2:** Univariate logistic regression of health economics in SAI patients.

Variables	SAI (*n =* 168) Median (P25, P75)/*N* (%)	NI (*n =* 668) Median (P25, P75)/*N* (%)	OR (95% CI)	*p*-value
Length of Hospital Stay (LOS), days	9 (7, 10)	8 (7, 9)	1.155 (1.081, 1.234)	0.000*
Hospitalization Costs, RMB	28114.04 (23230.12, 33379.85)	22292.84 (19203.53, 25999.63)	1.001 (1.001, 1.001)	0.000*

OR, odds ratio; CI, confidence interval. This figure presents the results of a one-way logistic regression analysis examining length of hospital stay (LOS) and hospitalization costs between SAI and NI patients. The analysis includes median values (M) and interquartile ranges (P25, P75) for both groups. The odds ratios (OR) with 95% confidence intervals (CI) and *p*-values are reported for each variable. Both LOS and hospitalization costs show significant differences between the two groups, with *p*-values of 0.000, indicating statistical significance. SAI, stroke-associated infection; NI, no infection. *p < 0.01.

### 3.3 Risk factors for SAI

#### 3.3.1 Comparison of baseline characteristics between cohorts

The baseline characteristics of patients in the training and validation cohorts are summarized in Table 1. The two cohorts were well balanced across demographic, clinical, and laboratory variables. These findings indicate that the training and validation cohorts were well matched.

#### 3.3.2 Univariate analysis of potential predictors

Univariate logistic regression identified following variables significantly associated with SAI (*p <* 0.05), including NIHSS at admission, mRS at admission, age, sex, smoking, alcoholism, and certain laboratory variables, including HCY, WBC, NEUT, NLR, PT, BUN, Ccr, AST, FT3, and TSH (Table 3). These variables, all of which had variance inflation factor values < 10, were entered into multivariate logistic regression to determine independent predictors of SAI.

**Table 3:** Univariate logistic regression for SAI predictors.

Variables	SAI (*n =* 168) Median (P25, P75)/*N* (%)	NI (*n =* 668) Median (P25, P75)/*N* (%)	B	Wald	OR (95%CI)	*P*-value
NIHSS at admittance	8 (3.25, 15)	3 (2, 6)	0.198	105.870	1.22 (1.17, 1.27)	0.000*
mRS at admittance	3 (1, 4)	1 (1, 2)	0.752	115.433	2.121 (1.85, 2.43)	0.000*
Age, years	77.5 (65, 84)	67 (59, 75.75)	0.059	50.194	1.061 (1.04, 1.08)	0.000*
Sex, no (%)						
Man	88	450	−0.629	12.917	0.533 (1.33, 2.65)	0.000*
Women	80	218				
Smoking (%)	33.3	43.4	−0.428	5.575	0.652 (0.46, 0.93)	0.018*
Alcoholism (%)	18.4	29.6	−0.622	8.266	0.537 (0.35, 0.82)	0.004*
Medical history (%)						
Atrial fibrillation	32.1	13.7	1.087	29.622	2.966 (2.00, 4.39)	0.000*
Arterial hypertension	76.7	77.9	−0.069	0.113	1.07 (0.72, 1.60)	0.736
Diabetes	35.1	26.7	0.391	4.533	1.479 (1.03, 2.12)	0.033*
Coronary heart disease	23.2	15.5	0.494	5.459	1.64 (1.08, 2.48)	0.019*
Dyslipidemia	30.3	40.2	−0.436	5.532	0.647 (0.45, 0.93)	0.019*
Previous stroke	20.2	11.3	0.681	8.897	1.976 (1.27, 3.09)	0.003*
Systolic blood pressure at admittance (mmHg)	161 (141, 175)	159 (144, 172)	−0.001	0.067	0.999 (0.99, 1.00)	0.795
Diastolic blood pressure at admittance (mmHg)	85 (77, 94)	87 (80, 95)	−0.009	1.428	0.991 (0.98, 1.01)	0.232
Stroke subtype based on TOAST						
Large-artery atherosclerosis	78	290	−0.009	0.017	0.991 (0.88, 1.13)	0.896
Cardioembolism	23	157				
Small-vessel occlusion	42	78				
Stroke of other determined etiology	15	112				
Stroke of undetermined etiology	10	31				
Location of responsible vessel						
Anterior circulation	116	466	0.044	0.131	1.045 (0.82,1.32)	0.718
Posterior circulation	30	132				
Anterior and posterior circulation	22	79				
Biochemical variables						
High density lipoprotein (HDL)	1.08 (0.91, 1.27)	1.04 (0.88, 1.22)	0.453	2.323	1.573 (0.88, 2.81)	0.127
Apolipoprotein A1 (Apo A1)	1.26 (1.12, 1.39)	1.25 (1.12, 1.39)	−0.185	0.217	0.831 (0.38, 1.81)	0.641
Apolipoprotein B (Apo B)	0.96 (0.78, 1.14)	0.94 (0.80, 1.09)	0.067	0.646	1.069 (0.91, 1.26)	0.421
Lipoprotein (a) (Lp (a))	185.5 (81, 329.5)	172 (78, 344.5)	0.000	0.121	1.000 (1.00, 1.00)	0.728
Homocysteine (HCY)	15.05 (12, 20.2)	13.2 (10.9, 16.5)	0.024	8.814	1.024 (1.01, 1.04)	0.003*
White blood count (WBC)	7.72 (6.02, 9.75)	7.04 (5.95, 8.58)	0.131	14.178	1.14 (1.07, 1.22)	0.000*
Neutrophils (NEUT)	4.84 (3.57, 6.85)	4.28 (3.49, 5.60)	0.160	18.481	1.173 (1.09, 1.26)	0.000*
Lymphocyte (LYM)	1.83 (1.23, 2.48)	1.93 (1.47, 2.54)	−0.079	0.710	0.924 (0.77, 1.11)	0.400
Neutrophil/Lymphocyte (NLR)	2.42 (1.67–4.40)	2.18 (1.56–3.33)	0.118	14.301	1.125 (1.06, 1.20)	0.000*
Prothrombin time (PT)	13.2 (13.6, 14.1)	13.0 (12.5, 13.5)	0.482	24.623	1.61 (1.34, 1.96)	0.000*
Activated partial thromboplastin time (APTT)	35.5 (32.7, 39.3)	35.6 (32.6, 39.4)	−0.008	0.269	0.992 (0.96, 1.02)	0.604
Fibrinogen	3.29 (2.86, 3.90)	3.25 (2.84, 3.69)	−0.007	0.139	0.993 (0.96, 1.03)	0.710
Glucose	7.37 (6.16, 9.49)	6.23 (5.62, 8.62)	0.040	3.210	1.04 (1.00, 1.09)	0.073
Blood Urea Nitrogen (BUN)	7.10 (5.49, 9.00)	6.13 (5.17, 7.43)	0.204	32.678	1.226 (1.14, 1.31)	0.000*
Creatinine clearance (Ccr)	67 (56, 83)	68 (57, 79)	0.008	9.143	1.008 (1.00, 1.01)	0.002*
Glomerular filtration rate (GFR)	93.98 (70.85, 112.89)	101.06 (84.15, 120.10)	−0.003	1.622	0.997 (0.99, 1.00)	0.203
Alanine aminotransferase (ALT)	16 (11, 22)	17 (13, 26)	−0.007	1.184	0.993 (0.98, 1.01)	0.277
Aspartate aminotransferase (AST)	19 (16, 24)	19 (15, 24)	0.015	5.405	1.015 (1.00, 1.03)	0.020*
Gamma-glutamyl transpeptidase (GGT)	22.00 (15.75, 38.25)	24.00 (17.00, 36.00)	−0.001	0.061	0.999 (0.99, 1.00)	0.805
Free triiodothyronine (FT3)	4.02 (3.48, 4.49)	4.51 (4.01, 5.02)	−0.189	7.387	0.828 (0.52, 0.81)	0.007*
Free thyroxine (FT4)	15.65 (13.55, 16.95)	15.09 (13.75, 16.63)	0.016	0.423	1.016 (0.98, 1.14)	0.515
Thyroid stimulating hormone (TSH)	1.05 (0.61, 1.65)	1.42 (0.81, 2.40)	−0.216	7.268	0.805 (0.67, 0.94)	0.007*
BMI	24.92 (22.79, 27.30)	25.07 (23.29, 27.34)	−0.017	0.527	0.983 (0.94, 1.03)	0.468

OR, odds ratio; CI, confidence interval. This figure presents the results of the univariate logistic regression analysis examining the predictors for SAI. The analysis includes variables such as NIHSS at admittance, mRS at admittance, age, sex, and various medical history factors. The unstandardized regression coefficients (B), Wald statistics, odds ratios (OR) with 95% confidence intervals (CI), and P-values are reported for each variable. Statistically significant predictors of SAI are indicated by *p* < 0.05. SAI, stroke-associated infection; NI, no infection. *p < 0.01.

#### 3.3.3 Multivariate logistic regression for independent predictors

Multivariate logistic regression was performed with variables above which were significant in the univariate analysis, and identified 5 factors as independent predictors for SAI, which included higher mRS at admission, male sex, prolonged PT, elevated BUN, and lower TSH (Table 4).

**Table 4:** Multivariate logistic regression for independent SAI predictors.

Variables	SAI (*n =* 168) Median (P25, P75)/*N* (%)	NI (*n =* 668) Median (P25, P75)/*N* (%)	B	Wald	OR (95%CI)	*P*-value
NIHSS at admittance	8 (3.25, 15)	3 (2, 6)	0.064	2.770	1.066 (0.989, 1.149)	0.096
mRS at admittance	3 (1, 4)	1 (1, 2)	0.308	4.666	1.361 (1.029, 1.801)	0.031*
Age, years	77.5 (65, 84)	67 (59, 75.75)	0.022	3.152	1.022 (0.998, 1.047)	0.076
Sex, no (%)						
Man	88	450	−0.745	5.952	0.475 (0.261, 0.864)	0.015*
Women	80	218				
Smoking (%)	33.3	43.4	0.012	0.001	1.012 (0.529, 1.934)	0.971
Alcoholism (%)	18.4	29.6	−0.121	0.118	0.886 (0.443, 1.772)	0.732
Biochemical variables						
Homocysteine (HCY)	15.05 (12, 20.2)	13.2 (10.9, 16.5)	0.009	0.418	1.009 (0.982, 1.036)	0.518
White blood count (WBC)	7.72 (6.02, 9.75)	7.04 (5.95, 8.58)	0.094	0.589	1.099 (0.864, 1.396)	0.443
Neutrophils (NEUT)	4.84 (3.57, 6.85)	4.28 (3.49, 5.60)	0.046	0.087	1.047 (0.771, 1.421)	0.769
Neutrophil/Lymphocyte (NLR)	2.42 (1.67, 4.40)	2.18 (1.56, 3.33)	−0.006	0.010	0.994 (0.881, 1.120)	0.920
Prothrombin time (PT)	13.2 (13.6, 14.1)	13.0 (12.5, 13.5)	0.361	7.514	1.435 (1.108, 1.858)	0.006*
Blood Urea Nitrogen (BUN)	7.10 (5.49, 9.00)	6.13 (5.17, 7.43)	0.144	4.981	1.154 (1.018, 1.310)	0.026*
Creatinine clearance (Ccr)	67 (56, 83)	68 (57, 79)	0.002	0.211	1.002 (0.992, 1.013)	0.646
Aspartate aminotransferase (AST)	19 (16, 24)	19 (15, 24)	0	0.001	1.002 (0.982, 1.017)	0.973
Free triiodothyronine (FT3)	4.02 (3.48, 4.49)	4.51 (4.01, 5.02)	−0.064	0.577	0.938 (0.795, 1.107)	0.448
Thyroid stimulating hormone (TSH)	1.05 (0.61, 1.65)	1.42 (0.81, 2.40)	−0.25	7.453	0.779 (0.650, 0.932)	0.006*

OR, odds ratio; CI, confidence interval. This figure presents the results of a multivariate logistic regression analysis for predicting SAI. The analysis includes variables such as NIHSS, mRS, age, sex, smoking, and biochemical indices. The unstandardized regression coefficients (B), wald statistics, odds ratios (OR) with 95% confidence intervals (CI), and *P*-values are provided for each predictor. Statistically significant predictors are indicated with *p* < 0.05. Significant predictors include age, sex, homocysteine, PT, and TSH. SAI, stroke-associated infection; NI, no infection. *p < 0.01.

#### 3.3.4 Multimodel logistic regression analysis

To further evaluate the robustness of the above 5 independent risk factors of SAI, multimodel logistic regression with progressive adjustments were performed (Table 5). Results showed that mRS at admission, sex, PT, BUN and TSH all remained independent significant predictors of SAI.

**Table 5:** Multimodal logistic regression for SAI predictors (progressive adjustment).

Variables	Model 1		Model 2		Model 3	
	OR (95%CI)	*P*-value	OR (95%CI)	*P*-value	OR (95%CI)	*P*-value
NIHSS at admittance	1.19 (1.15–1.24)	<0.001*	1.19 (1.14–1.24)	<0.001*	1.05 (0.98–1.14)	0.181
mRS at admittance	1.97 (1.71–2.28)	<0.001*	1.95 (1.68–2.25)	<0.001*	1.46 (1.09–1.95)	0.011*
Age, years	1.06 (1.04–1.08)	<0.001*	1.06 (1.04–1.08)	<0.001*	1.02 (1.00–1.05)	0.086
Sex, no (%)						
Man						
Women	1.62 (1.03–2.53)	0.035*	1.73 (1.09–2.73)	0.019*	2.85 (1.48–5.51)	0.002*
Smoking (%)						
No						
Yes	0.70 (0.43–1.16)	0.168	0.67 (0.40–1.12)	0.127	0.95 (0.48–1.88)	0.882
Alcoholism (%)						
No						
Yes	1.23 (0.72–2.09)	0.452	1.24 (0.73–2.13)	0.43	0.89 (0.43–1.87)	0.759
Biochemical variables						
Homocysteine (HCY)	1.03 (1.01–1.05)	0.004*	1.03 (1.01–1.05)	0.006*	1.01 (0.97–1.04)	0.714
White blood count (WBC)	1.17 (1.09–1.26)	<0.001*	1.17 (1.09–1.26)	<0.001*	1.08 (0.85–1.38)	0.514
Neutrophils (NEUT)	1.19 (1.10–1.28)	<0.001*	1.18 (1.10–1.28)	<0.001*	1.06 (0.78–1.43)	0.715
Neutrophil/Lymphocyte (NLR)	1.10 (1.04–1.18)	0.002*	1.10 (1.03–1.17)	0.002*	0.96 (0.87–1.07)	0.473
Prothrombin time (PT)	1.42 (1.16–1.74)	<0.001*	1.42 (1.15–1.74)	<0.001*	1.39 (1.05–1.85)	0.022*
Blood Urea Nitrogen (BUN)	1.16 (1.08–1.25)	<0.001*	1.16 (1.07–1.24)	<0.001*	1.17 (1.05–1.30)	0.003*
Creatinine clearance (Ccr)	1.01 (1.01–1.02)	0.004*	1.01 (1.01–1.02)	0.006*	1.00 (0.99–1.02)	0.438
Aspartate aminotransferase (AST)	1.02 (1.01–1.03)	0.008*	1.02 (1.01–1.03)	0.01*	1.00 (0.98–1.02)	0.987
Free triiodothyronine (FT3)	0.82 (0.64–1.06)	0.123	0.84 (0.66–1.07)	0.161	0.93 (0.73–1.20)	0.585
Thyroid stimulating hormone (TSH)	0.74 (0.62–0.89)	0.001*	0.75 (0.63–0.90)	0.002*	0.76 (0.63–0.93)	0.006*

OR, odds ratio; CI, confidence interval Model 1: Adjust: age, sex, smoking, alcohol use, arterial hypertension, diabetes, dyslipidemia. Model 2: Adjust: age, sex, smoking, alcohol use, arterial hypertension, diabetes, dyslipidemia, coronary heart disease, previous stroke. Model 3: Adjust: age, sex, smoking, alcohol use, arterial hypertension, diabetes, dyslipidemia, coronary heart disease, previous stroke, NIHSS at admittance, mRS at admittance, homocysteine (HCY), white blood count (WBC), neutrophils (NEUT), neutrophil/lymphocyte (NLR), prothrombin time (PT), blood urea nitrogen (BUN), Creatinine clearance (Ccr), aspartate aminotransferase (AST), free triiodothyronine (FT3), thyroid stimulating hormone (TSH). This figure presents the results of multimodal logistic regression analysis examining predictors for SAI across three progressively adjusted models. Model 1 adjusted for age, sex, smoking, alcohol, arterial hypertension, diabetes, and dyslipidemia. Model 2 additionally adjusted for coronary heart disease and previous stroke. Model 3 further adjusted for NIHSS at admittance, mRS at admittance, HCY, WBC, NEUT, NLR, PT, BUN, Ccr, AST, FT3, and TSH. The odds ratios (OR) with 95% confidence intervals (CI) and *p*-values are reported for each predictor. Statistically significant results are indicated by *p* < 0.05. SAI, stroke-associated infection. *p < 0.01.

### 3.4 Development and validation of a predictive nomogram for SAI

**Figure 3:**
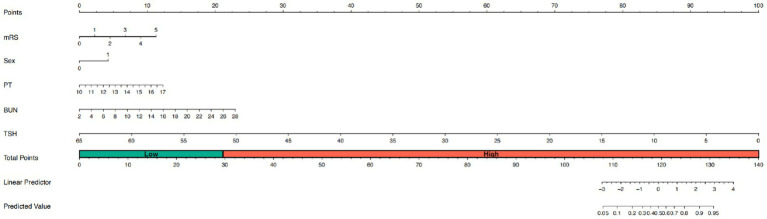
Nomogram for SAI prediction in rt-PA-treated AIS patients. This figure presents the nomogram developed to predict SAI in rt-PA-treated AIS patients. The nomogram incorporates several predictive factors, including mRS at admittance, PT, BUN, and TSH. For each variable, a corresponding point is assigned on the top point scale. The sum of these points yields a total points value, which is directly mapped to the predicted probability of SAI on the bottom probability scale. For clinical convenience, the total point range is divided into low risk and high risk zones (indicated on the figure), allowing immediate risk stratification at the bedside without further calculation. mRS, mRS at admittance; PT, prothrombin time, BUN, blood urea nitrogen; TSH, thyroid stimulating hormone.

**Figure 4:**
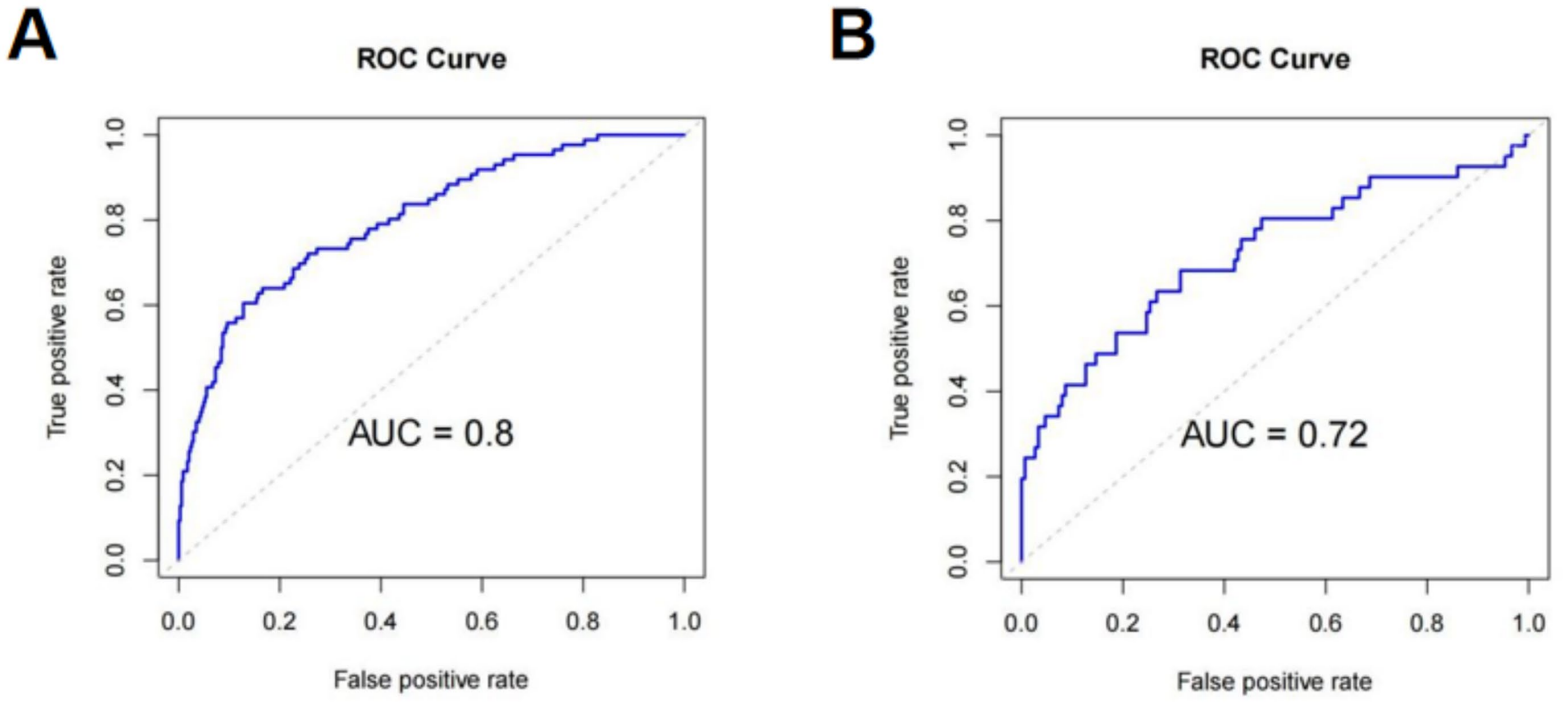
ROC curves of SAI nomogram: Training **(A)** and validation **(B)** cohorts. ROC curves are plotted with sensitivity (true positive rate; *y*-axis, 0–1) on the ordinate and 1-specificity (false positive rate; *x*-axis, 0–1) on the *x*-axis, with the diagonal representing no discriminative ability (AUC = 0.5). **(A)** Training cohort (*n =* 586), AUC = 0.80, indicating good SAI case identification ability. **(B)** Validation cohort (*n =* 250), AUC = 0.72, indicating acceptable performance; the slight decrease may be due to sample size or variability. ROC: receiver operating characteristic, AUC: area under the curve.

**Figure 5:**
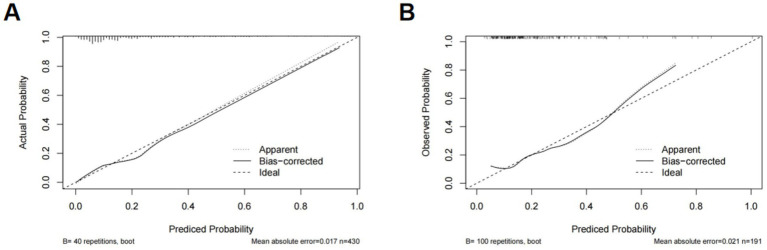
Calibration curves of SAI nomogram: Training **(A)** and validation **(B)** cohorts. The calibration plot compares the predicted probability (*x*-axis, 0–1) with the observed probability (*y*-axis, 0–1), with an ideal 45-degree line indicating perfect consistency. **(A)** Training cohort (*n =* 586), showing the apparent (uncorrected) curve, the bias-corrected curve (adjusted for overfitting via 1,000 bootstrap resampling), and the ideal curve; mean absolute error = 0.017; Hosmer-Lemeshow goodness-of-fit test *p* = 0.811 (*p* > 0.05 = no significant calibration error). **(B)** Validation cohort (*n =* 250), curves similar; mean absolute error 0.021. Points close to the ideal line reflect accurate predictions; minimal bias confirms its reliability.

Using the 5 independent predictors, a nomogram was developed to estimate the probability of SAI in AIS patients receiving rt-PA thrombolysis (Figure 3). Each predictor contributes points summed to generate an individual total score, corresponding to the predicted risk. The nomogram demonstrated good discrimination, with an AUC of 0.80 (Figure 4A), and satisfactory calibration, confirmed by the calibration curve and Hosmer-Lemeshow test (*p* = 0.811; Figure 5A). Internal validation using bootstrap resampling yielded consistent predictive performance (AUC 0.72; Figure 4B) and calibration (Figure 5B).

## 4 Discussion

This study comprehensively characterized SAI following intravenous thrombolysis in AIS patients and developed a nomogram-based prediction model. Five independent risk factors were identified: higher mRS at admissionore, male sex, prolonged PT, elevated BUN, and lower TSH. Unlike previous models focused primarily on stroke-associated pneumonia (Huang et al., 2019; Zhang et al., 2021; Wang et al., 2023), this study expands the prediction target to encompass the full spectrum of SAI, including pneumonia, urinary tract infection, upper respiratory tract infection, gastrointestinal infection, and less common sites such as periodontal, conjunctival, and cutaneous infections (Rocco et al., 2007). This comprehensive approach better reflects the systemic nature of post-stroke immunodepression and provides a more clinically relevant framework for infection risk assessment. Furthermore, by integrating routinely available clinical and laboratory parameters into an intuitive nomogram, this model offers a practical tool for early risk stratification that can inform targeted monitoring and preventive interventions in the hyperacute stroke setting.

The identification of higher admission mRS as a predictor of SAI is consistent with extensive evidence linking functional impairment to stroke-induced immunodepression (Westendorp et al., 2011; Chamorro et al., 2007). Severe neurological deficits compromise multiple protective mechanisms: impaired swallowing increases aspiration risk, reduced mobility promotes venous stasis and pressure ulcers, diminished cough reflex facilitates airway colonization, and autonomic dysregulation disrupts immune homeostasis (Meisel and Meisel, 2011; Finlayson et al., 2011). Importantly, recent investigations demonstrate that stroke severity directly correlates with the magnitude of systemic immunosuppression, as evidenced by lymphopenia, monocytic deactivation, and attenuated inflammatory responses (Prass et al., 2003; Chamorro et al., 2012). These converging lines of evidence support the biological plausibility of mRS as a robust predictor and underscore the importance of early functional assessment in infection risk stratification.

The increased SAI susceptibility in male patients aligns with emerging recognition of sex-specific immune responses following cerebral ischemia. Females generally exhibit more robust innate and adaptive immune activation, mediated partly through estrogen signaling and X-linked immune regulatory genes, whereas males demonstrate greater vulnerability to post-stroke immunodepression (Bravo-Alegria et al., 2017; Iadecola and Anrather, 2011). Mechanistic studies reveal sex differences in microglial activation patterns, T-cell trafficking, and cytokine profiles following ischemic injury in both experimental models and clinical populations (Anrather and Iadecola, 2016; Silva et al., 2023). Epidemiological analyses consistently report higher infection rates among male stroke survivors across diverse cohorts, independent of stroke severity and comorbidity burden (Reeves et al., 2008; Han et al., 2022). These findings provide both mechanistic rationale and empirical support for incorporating sex into infection risk prediction models and suggest potential value in sex-tailored prevention strategies.

The association between prolonged PT and SAI reflects the intricate interplay between coagulation activation and immune dysfunction in acute stroke. Prolonged PT may indicate impaired hepatic synthetic function, consumption coagulopathy, or systemic inflammatory stress—all conditions that compromise immune competence (del Zoppo and Gorelick, 2010; Kannemeier et al., 2007). The concept of “immunothrombosis” has emerged to describe the bidirectional crosstalk between hemostatic and immune systems, wherein coagulation proteases directly modulate inflammatory cell behavior and endothelial barrier function (Engelmann and Massberg, 2013). In the context of thrombolysis, tissue plasminogen activator may further perturb this balance through effects on the neurovascular unit, potentially exacerbating blood–brain barrier disruption and enhancing infection vulnerability (Liu et al., 2023; Miller et al., 2011). Large registry studies have documented associations between coagulation abnormalities and adverse outcomes including infectious complications following thrombolytic therapy (Wardlaw et al., 2014), supporting the clinical relevance of PT as a risk marker.

Elevated BUN emerged as a strong metabolic predictor, consistent with its established role as an integrative biomarker reflecting renal function, hydration status, and catabolic stress (Ugajin et al., 2012; Aronson et al., 2004). BUN elevation signals activation of neurohormoral pathways that impair immune resilience, including sympathetic nervous system activation and renin-angiotensin-aldosterone system upregulation (Kazory, 2010). The kidney-brain axis concept provides a unifying framework linking renal dysfunction to cerebrovascular disease burden through shared vascular vulnerabilities (Nam et al., 2024). Beyond impaired clearance, elevated BUN may reflect enhanced protein catabolism and metabolic derangement that directly compromise immune function in critically ill patients (Faura et al., 2021). Similarly, reduced TSH highlights the importance of neuroendocrine dysregulation in post-stroke infection susceptibility. Low TSH characterizes non-thyroidal illness syndrome (NTIS), reflecting hypothalamic–pituitary suppression during acute critical illness (Suda et al., 2018b; O'Keefe et al., 2015; Wu et al., 2025). Stroke-specific investigations have linked low FT3 levels to both mortality and post-stroke infections, suggesting thyroid hormone alterations serve as both illness severity markers and potential mechanistic contributors to immune dysfunction (Suda et al., 2016; Taroza et al., 2020). Recent evidence indicates that thyroid hormone status influences neutrophil function, T-cell proliferation, and cytokine production, providing biological mechanisms linking NTIS to infection risk (Bunevicius et al., 2015; Liu et al., 2018). Collectively, these metabolic and endocrine markers capture dimensions of physiological stress that complement traditional neurological severity assessments.

The clinical utility of the nomogram extends beyond mere risk prediction to support actionable decision-making in the acute stroke setting. Early identification of high-risk patients enables targeted implementation of evidence-based preventive measures, including intensified monitoring protocols, optimized oral care and swallowing assessment, early mobilization strategies where feasible, and judicious antimicrobial stewardship (Smith et al., 2015; Klehmet et al., 2009). Risk stratification can inform resource allocation decisions, such as level of care assignment and intensity of nursing surveillance, potentially improving efficiency of care delivery in resource-constrained environments (Katzan et al., 2007). Moreover, the model’s integration of routinely obtained admission variables facilitates rapid assessment without requiring specialized testing or delays, making it particularly suited for implementation in time-sensitive thrombolysis workflows (Su et al., 2025). Future investigations should evaluate whether nomogram-guided care protocols can reduce SAI incidence, shorten hospital stays, and improve functional outcomes compared to standard care approaches. The potential for this tool to support precision medicine approaches in stroke care—wherein preventive interventions are tailored to individual risk profiles—represents an important avenue for translating predictive models into improved patient outcomes (Cao et al., 2025; Powers et al., 2019).

Several limitations warrant acknowledgment. First, as a single-center prospective cohort study, external validation in geographically and demographically diverse populations is essential to confirm generalizability and assess performance across different healthcare settings with varying infection surveillance practices and antimicrobial stewardship protocols. However, the large sample size, comprehensive phenotyping, and use of routinely collected variables enhance the likelihood of model transportability. Second, the study did not incorporate dynamic biomarkers measured serially during hospitalization, such as C-reactive protein trajectories, procalcitonin kinetics, or longitudinal immune cell profiling, which might further enhance predictive accuracy. Nonetheless, the model’s reliance on admission variables provides practical advantages for early risk stratification before such dynamic markers become available. Third, while traditional logistic regression offers interpretability advantages and facilitates clinical implementation through nomogram visualization, emerging machine learning approaches might capture complex nonlinear relationships and interactions among predictors. Future work incorporating advanced algorithms with appropriate validation strategies could potentially improve discrimination, though interpretability and clinical acceptability must be carefully balanced against incremental performance gains. Fourth, the study did not assess the impact of specific preventive interventions or infection control measures on SAI occurrence, limiting causal inference regarding modifiable factors. Prospective implementation studies are needed to determine whether nomogram-guided care protocols influence infection rates and outcomes. Finally, long-term outcomes including disability, quality of life, and economic burden were not evaluated. Despite these limitations, this study provides a clinically relevant tool addressing an important gap in post-thrombolysis care and establishes a foundation for future validation and implementation research.

## 5 Conclusion

A comprehensive profile of SAI following intravenous rt-PA thrombolysis was delineated, including the distribution of infection types and their clinical characteristics. Five independent risk factors—mRS, sex, PT, BUN, and TSH—were identified as contributors to SAI risk. Based on these parameters, a nomogram was constructed to enable early prediction of SAI in individuals with acute ischemic stroke undergoing thrombolysis. This tool enables early risk stratification, facilitates targeted preventive strategies, and has the potential to reduce infection incidence, hospitalization duration, and associated healthcare burden. Future studies are warranted to externally validate the model and assess its impact on clinical outcomes.

## Data Availability

The raw data supporting the conclusions of this article will be made available by the authors, without undue reservation.
